# Quality of urban parks in the perception of city residents with mobility difficulties

**DOI:** 10.7717/peerj.10570

**Published:** 2020-12-18

**Authors:** Magdalena Błaszczyk, Marzena Suchocka, Magdalena Wojnowska-Heciak, Magdalena Muszyńska

**Affiliations:** 1 Department of Landscape Architecture, Institute of Environmental Engineering, Warsaw University of Life Sciences - SGGW, Warsaw, Poland; 2 Unaffiliated, Warsaw, Poland

**Keywords:** Park accessibility, Park availability, Social survey, Blind, Vision impaired, Carers/assistants of people with a disability, People who use a wheelchair, Parents of children who use strollers to navigate in urban park, Limited mobility park users

## Abstract

Urban parks should be inclusive for all. Availability and accessibility of urban parks determine the quality of life in cities. The importance of access increases for residents with limited mobility who, facing obstacles due to inadequate adjustment of the surrounding physical space, are exposed to social exclusion. Five groups of respondents completed a survey questionnaire revealing their attitudes towards green areas and indicating barriers to parks’ accessibility. The groups were designed to include blind and vision impaired people, those who use a wheelchair, have a physical disability of any kind, their carers/assistants and parents pushing strollers. The results revealed more similarities than differences among the five groups (the differences included preferences towards the neighbourhood and destination parks, physical barriers in parks, as well as using assistive technology devices and mobile assistive applications). Overall, city residents with mobility difficulties find those green public spaces as an important element of their life quality.

## Introduction

Urban parks play a key role in determining the quality of life of city dwellers, performing natural, economic, aesthetic, social, and health functions (Humpel, 2002; Bedimo-Rung, Mowen & Cohen, 2005; Byrne & Wolch, 2009; Ulrich & Addoms, 1981; Wolch, Byrne & Newell, 2014). The presence of open spaces (especially neighbourhood parks) can combat many urban diseases, relieve public stress and help build welcoming and inclusive neighbourhoods (Bedimo-Rung, Mowen & Cohen, 2005; Wolch, Byrne & Newell, 2014; Wang, 2015). Proximity to green spaces and high visit frequency is associated with a perceived mental well-being improvement and better physical health condition (Romagosa, 2018; Houlden et al., 2019; Liu et al., 2019; Martin et al., 2020). People who are deprived of contact with nature or have difficult access to green areas are more at risk of depression and decreased functional ability, which in turn can lead to a significant deterioration in the quality of their life (Whyte, 2001; Xie et al., 2019). That is why it seems essential for green areas in the urban agglomeration to be safe, available, and accessible to everyone, as for many people spending time in the park is the only possibility of close contact with nature in everyday life. Park accessibility (and availability) seems to be particularly important to people with a disability and people with mobility difficulties, offering an alternative environment for rehabilitation and mitigation of health disparities (Saitta et al., 2019). Very often, however, access to park spaces is made significantly more difficult due to the presence of many architectural (Perry et al., 2018a) social or mental barriers (Kimic, Maksymiuk & Suchocka, 2019; Suchocka, Kimic & Widaj, 2019).

### Availability, accessibility and park use

Both availability and accessibility determine park use to some extent. While the term “park availability” applies to its existence in an urban space measured using, for example, the walking distance (Giles-Corti et al., 2005), “accessibility” generally refers to the presence of different barriers (Park, 2017). The barriers can be recognised at physical and psychological levels, and measured, for instance, through the aspect of safety (Biernacka & Kronenberg, 2018). Many studies suggest that higher accessibility leads to higher park use (Scott & Jackson; McCormack et al., 2010; Zhang & Zhou, 2018) and that proximity to parks and other urban green spaces (availability) increases people’s willingness to visit them (Bedimo-Rung, Mowen & Cohen, 2005; Byrne & Wolch, 2009; Giles-Corti et al., 2005; Reyes, Páez & Morency, 2014; Liang, Chen & Zhang, 2017; Wüstemann, Kalisch & Kolbe, 2017; Feng et al., 2019). However, some studies proved that accessibility and availability might not influence people’s preferences and frequency of visits (Hillsdon et al., 2006; Payne, 2002), indicating other factors, such as time (Lin et al., 2014) and people’s approach to nature to be the most important for park use (Wang, 2015; Lin et al., 2014). According to Wang, conventional urban open space planning that relies on objective indices such as area and number of parks per capita to measure people’s access to urban parks does not contain all contributing to the general perception of the accessibility factors. The accessibility concept and decision-making processes seem to be more complicated (Wang, 2015).

### Physical dimensions of accessibility and availability

It has been recognised that the concept of accessibility should include some qualitative attributes such as the convenience or ease of overcoming distances (i.e., transport availability) (Penchansky & Thomas, 1981; Larkin & Peters, 1983). Two consecutive editions of the Dictionary of Human Geography (4th edition in 2005 and 5th edition in 2009) address accessibility as a broader concept that requires increasing conceptual emphasis on non-spatial mismatch. In the fourth edition (2005), the standard definition of accessibility is read as the ease with which one place can be reached from another, which broadens the concept to include interactions with other places and services, transport and communication constraints, and other socioeconomic barriers (Gregory et al., 2005). In addition to physical variables, Wang et al. (2015) proposes the non-physical dimension that plays a role when considering access to urban parks.

### Non-physical dimensions of accessibility and availability

According to Penchansky & Thomas (1981), an individual’s perception of accessibility may diminish or promote the use of urban facilities or services (e.g., parks) through behavioural choices. People’s judgement about which place or service is more accessible in comparison to other places initiates an integrated evaluation process (Penchansky & Thomas, 1981). Researchers describe “socio-organisational accessibility” to distinguish non-spatial factors from geographic factors, including social structures and mechanisms like cultural, ethnic, economic, and demographic attributes (Andersen & Aday, 1978; Ferreira & Batey, 2007). It has been found that there is a kind of the knowledge dimension influencing the accessibility of an urban park. It refers to the level of information that individuals have. It connects people’s subjective impressions about the place (Ferreira & Batey, 2007) like facilities in parks and activities held in parks.

Moreover, there are also social and personal dimensions that impact park accessibility (Andersen & Aday, 1978; Marten & Gillespie, 1978). High levels of interaction within a community improve social cohesion (Pirie, 1979). Urban parks offer opportunities for contact irrespective of non-spatial factors associated with socioeconomic constraints and personal capacities (i.e., health status, lifestyle, stage of life).

### Perceived accessibility versus place accessibility

The place accessibility directly correlates with levels of park safety, maintenance, attractiveness, opportunities for socialisation, and neighbourhood crime safety, aesthetics, quality of materials used in the park. Safety and opportunity for socialisation are one of the most important factors that positively relate to the monthly frequency of visits to an urban park (Leslie, Cerin & Kremer, 2010). Other objective indicators include also travel times, distances, place characteristics (Morris, Dumble & Wigan, 1979) and age, physical mobility, income, vehicle access of the park users (Combs et al., 2016). Both park-based and user-based factors may affect people’s perception of park access and park use (Byrne & Wolch, 2009). Perceived park access can be explained by park-based factors (internal features that operate within park areas), including lighting, signage, locations of facilities, program and activities, landscape design, and maintenance frequency (Gobster, 1998). It relates to how people rate the condition in which they live (Lättman, Olsson & Friman, 2020). Perception of the park can vary individually and change depending on, for instance, time of departure (Scott et al., 2007) and traffic. Perceived accessibility affects the park use as the attitude has a strong influence on park demand (Morris, Dumble & Wigan, 1979; Zhang & Tan, 2019; Wang et al., 2015).

### Park users with limited mobility

The dominating group of park-users with mobility problems are persons with a disability. Disabilities directly affecting movement include physical disabilities and visual impairment. Estimated 15% of the world’s population lives with a disability (World Health Organization, 2011). In Poland, 4.9 million people have a disability certificate, and the total number of people with a disability may reach 7.7 million (Central Statistics Office of Poland (GUS), 2016). It can be expected that due to the ageing of the population, these numbers will increase. Definition of disability proposed by WHO (World Health Organization) considers it a three-dimensional term covering impairment, activity limitations, and participation restrictions, reflecting the interaction between features of a person’s body and features of the society in which he or she lives (World Health Organization, 2011). According to researchers of the social model of disability, it is often the environment, not the medical impairment that acts as an agent of disability; therefore, the term “limited mobility” park-users covers not only people with a physical disability (Gronvik, 2007). Mobility difficulties and lack of sufficient physical activity are a common problem among seniors (Hirvensalo, Rantanen & Heikkinen, 2000; Yale School of Medicine, 2020), but limited mobility is not only a problem of old age. It may be a temporal situation in which a particular person finds herself/himself (Iezzoni et al., 2001). Groups that often assist people with a disability are carers or assistants. They share with their dependants a joint fight to overcome physical obstacles in urban space (pushing wheelchairs, etc.). Another group facing mobility difficulties are parents navigating with strollers in the urban park. They experience similar stress as people with a disability (Oh, Lee & Park, 2015; Currie & Develin, 2010; Inoue, Baker & Scott, 2009).

It is critical to understand for whom accessibility is being defined (Wang, 2015). The most important aspect for people with limited mobility is the possibility of pedestrian circulation. Accessibility must be considered as an essential feature of the human-centred design in the built environment (Yılmaz, 2018). According to this approach, called in some studies ’universal design’, discrimination should be eliminated. Inclusive and egalitarian approach refers to space and product accessibility. The concept of universal design helps to understand vulnerable people better. Those with limited mobility (older people, people with a physical disability, people with an intellectual disability or mental illness, children, mothers and the like) are valued and empowered. This kind of inclusive approach requires accepting that all the people are equal in the built environment (Yılmaz, 2018; Arvanitis, 2004; Perry et al., 2018b).

### Aim of the study

Looking at the accessibility of urban parks, we focused on detecting physical discomfort while navigating in the studied areas (quality of pavements, location of benches, accessibility of restrooms, presence of equipment that facilitates entering the steps, etc.). Our target group covered the most sensitive detector of any obstacles in the space—people with limited mobility. The groups were asked questions about various aspects concerning urban park use and infrastructure. The study aimed to verify whether urban parks in Warsaw are perceived as accessible for city residents with limited mobility and to detect obstacles in navigating in urban parks in Warsaw. Working as landscape architects, we still look for the solutions to design in harmony with natural processes (e.g., considering rainwater infiltration) and to design for people (e.g., ensure that pavements would be easy to navigate for people who use a wheelchair). Therefore, it is of utmost importance to verify whether park users positively value the infrastructure used in green spaces. The issue of universal design of urban parks in Poland is rarely undertaken. Research concerning the perception of urban parks in Warsaw, Poland by people with limited mobility is scarce, and our study aimed to fill this knowledge gap.

## Materials & Methods

### Participants

The study population covers people who may encounter architectural barriers or other difficulties in moving around the park space due to:

a. own motor or sensory disability (e.g., vision) requiring the use of a wheelchair, crutches, canes, etc.

b. the motor or sensory disability of the dependant requiring the use of a wheelchair, crutches, canes, etc.

For the described problems, it is important to encounter physical barriers while moving, not the original cause of the mobility limitation. Therefore, people with other disabilities if they have mobility difficulties were included in the sample. Controlling disabilities other than motor or sensory disabilities affecting movement does not meet the study population definition criterion and does not fall within the scope of the studied problems. It is not only about intellectual disability, but also sensory disabilities that do not have a significant impact on motor skills (e.g., deafness). On the other hand, the population includes people with coexisting disabilities, or, for example, caregivers of children or adults requiring wheelchair mobility (including those with intellectual disabilities). Therefore, five groups of survey participants were involved in our study. All participants had some limitations in mobility (including physical impairment or blindness) or had to use equipment to navigate in urban public spaces (carers/assistants of people with a disability, parents who use strollers for their children to walk in the park). The first and second group covered blind and vision impaired. There are various terms used to describe different levels of vision impairment and blindness. In this study, blindness is defined as the state of being sightless. A blind individual is unable to see as it is the condition of total blackness of vision with the inability of a person to distinguish darkness from bright light in either eye. Under the term vision impaired, we understand that persons have partial vision, either in one or both eyes or low vision in which visual acuity is 20/70 or poorer in the better-seeing eye and cannot improve with glasses or contacts. The third group covered people with a physical disability related to orthopaedic issues (dominating group covered people who use a wheelchair). The research also included parents walking in the parks with their children in strollers. In their everyday living, they face the same problems as their dependents (Ren, 2020). The inclusion of carers/assistants assumed that disability is also associated with the relationship between the human condition and the surrounding environment (physical, social). People with a physical disability, carers/assistants of people with a physical disability and parents who use strollers for their children (Ren, 2020) face similar obstacles while navigating the city and urban parks.

The study sample was selected on purpose with the use of recruitment questions verifying belonging to the population groups described as above. Due to the lack of a population list, it is not possible to take a random sample. The lack of description of the population structure makes it difficult to select a proportional sample. However, a mix of representative different population groups that struggle with mobility difficulties has been applied. Considering, the data obtained from the Central Statistical Office in 2019 in Warsaw, there were 102,900 residents aged 0–4 in Warsaw in 2019; this number of parents needed a stroller to navigate in urban space (Central Statistics Office of Poland (GUS), 2019). At that time, every fourth resident of Mazovian voivodship had a certificate of disability; therefore, in Warsaw, there might live about 425,000 residents with a disability (Central Statistics Office of Poland (GUS), 2020). Unfortunately, there is no data concerning the current number of carers/assistants of people with a disability. Considering the number of people with mobility problems and Warsaw’s population of 1,700,000 residents, we may assume that almost half of Warsaw residents struggle with limited mobility.

The study group of 103 participants, included 58 women (56.3%), and 45 men (43.7%). The respondents were divided into five groups: people who use a wheelchair (29.2%), people with impaired physical mobility (19.4%), blind people (16.5%), vision-impaired park users (17.5%), and carers/assistants of people with a physical disability, representing 21.4% of the respondents. More than half of the respondents were young people aged 40 years or less. The largest group of respondents was that consisting of participants between 18 and 29 years of age (almost 31.1% of the respondents), followed by people aged 30–39 years (25.2%), 40–49 years (20.4%), 50–59 years (12.6%), and finally by people over 60 years old (10.7%). In terms of education, participants who completed higher and secondary education (in total, over 78.7% of the respondents) were the dominant group. The least numerous were those who completed only a basic cycle of primary school. The participants were all city dwellers. The detailed characterisation of the respondents is presented in Table 1.

**Table 1:** Sociodemographic characteristics of 103 participants of the survey.

**Gender**	Women	56.3%	**Age**	18–29 years	31.1%
				30–39 years	25.2%
	Men	43.7%		40–49 years	20.4%
				50–59 years	12.6%
				>60 years	10.7%
**Education**	Basic	2.9%	**Type of disability**	Wheelchair users	28.2%
	Vocational	13.6%		People with impaired mobility	18.4%
	High school	34.0%		Blind people	16.5%
	Higher	44.7%		Partially sighted	17.5%
	Students	4.8%		Carers of disabled adults and parents of children	19.4%

### Questionnaire

The questionnaires used in the study consisted of 13 questions. Five of them related to time spent in parks by people with a disability, their caregivers/assistants and parents who use strollers for their children to navigate inside the park) and their preferences, two concerned park accessibility, three –barriers and facilities, five –activities and comfort of movement. Closed single-choice answers dominated. One question allowed a maximum of three answers. Depending on the question, the respondents could choose between 3 and 10 answers, sometimes being allowed to provide their answers. A 5-point Likert scale was used to answer five questions, anchored by 1 and 5, and “Absolutely yes” and “Definitely no”.

### Survey

A pilot study conducted on 5 June 2019 helped verify research assumptions, the accuracy of the questions and their understanding. It took place in front of the entrance to the building of the District Disability Assessment Team in Warsaw at 5 Gen. Andersa Street in Warsaw. The survey began in June 2019 with the publication of the questionnaire using Google’s Worksheet on social networking sites dedicated to people with a disability, carers/assistants of people with a disability, seniors and parents walking with children in strollers. The questionnaires were distributed online according to the snowball method, starting from the invitations sent out via platforms, portals, websites dedicated to disabled people, seniors, young parents. More than 400 invitations to take part in the survey were sent as a link to the Google form. The survey response rate was approximately 30%. About 5–10% of completed forms were rejected due to inconsistent or too hasty responses. The whole process yielded a sample of 103 participants. The survey ended in August 2019.

### Statistical data analysis

Several methods were used to statistically analyse the survey data. The independence test *χ*2 was used to evaluate the differences between nominal variables. Analysis of the Likert scale data was performed with the Kruskal–Wallis one-way analysis of variance test. The results of multiple-choice questions were studied by correspondence analysis. All analyses were performed using Statistica 13.3.

## Results

### Visit duration and preferences for spending time in parks

The majority of respondents liked (definitely yes, rather yes) spending time in parks; nearly 11% did not like this activity. All respondents in the carers/assistants group liked spending time in this way, with 70% replying definitely yes and 30% rather yes. Only one vision impaired person used the rather not option. In the blind people group, more than 40% liked park visits. There were no significant statistical differences between the five groups of respondents.

In response to the question about frequency of park visits, around one-third of wheelchair users declared several to over a dozen visits a year, and the same percentage declared visiting parks several times a month. In the same group, 20% of the respondents declared spending time in the park more often than once a week. Among people with a disability, one-fifth declared daily visits. As for carers/assistants of people with a disability and parents with children in strollers, 50% declared several visits a week and 25% reported visiting the park every day. In the blind people group, only one person did not go to the park at all. Almost a quarter of blind respondents indicated that they spent time in the park every day, with nearly 30% visiting a park several times a month. A total of 18% of the whole surveyed population declared visiting a park every day, 33% several times a week, 22% several times a month, and 25% several to over a dozen times a year. Again, there were no significant statistical differences between the five groups of respondents.

More than half of the respondents usually spent about an hour in the park. Nearly 30% reported 2 and 3 h, and around 15% about 30 min. Nearly one per cent of the surveyed group declared a park visit of more than 3 h long. Among people who use a wheelchair, only 14% spent up to 30 min in the park. More than 57% of the respondents spent about 1 h in the park, 26% spent 2 to 3 h, and only 16% spent about 30 min in this way. Seventy per cent of the carers/assistants declared about one-hour long visits, and only one person admitted spending up to 30 min in the park. All remainder in this group spent 2 to 3 h in the park. A similar percentage was obtained in the vision impaired group. More than 66% of them spent about an hour, while a further 11% reported spending up to 30 min in the park. One person declared 3 hours’ visits. Almost the same number of blind people spent 30 min, about an hour, and about 2 to 3 h in the park. None of them was in the park for more than three hours. No significant statistical differences among the five groups of respondents were found.

Almost half (44%) of the respondents spend their time in both types of parks (destination and neighbourhood parks) but visit the local parks more often. Thirty-three per cent of the respondents chose neighbourhood parks only. The same percentage of people surveyed (around 11%) replied “I spend time in both types of parks, but more often in a destination park” and “I spend the same amount of time in a destination park as in a neighbourhood park”. Only one person out of all respondents chose only a destination park. Forty-one per cent of people who use a wheelchair spent time in both parks, but more often they chose a local one. Almost one-third of this group declared that they spent time only in a neighbourhood park. In the group of people with a disability, almost 58% visited the neighbourhood park only. In the group of carers/assistants, 90% declared spending time in a neighbourhood park (only or most often). Among the blind respondents, 35% liked to spend time in both parks but visited local parks more often. More than 23% of them spent the same amount of time in both types of parks. Among the partially sighted respondent, a neighbourhood park was the preferred option. There were significant statistical differences between the five groups of respondents (Table 2).

**Table 2:** Differences in the preferences for spending free time in the park depending on its scale: destination park (e.g., Royal Łazienki Park, Wilanów Palace and Park) versus neighbourhood park (e.g., parks close to home, pocket parks).

	I only spend time in destination parks	I spend time in both types of parks, but more often of in destination ones	I only spend time in neighbourhood parks	I spend time in both types of parks, but more often in neighbourhood ones	I spend as much time in both types of parks	*χ*2	df	*p*-value
People who use w wheelchair	0.97	4.85	8.74	11.7	1.94	39.91382	20	0.00512
People with impaired mobility	0.00	1.94	10.68	5.83	0.00			
Blind	0.00	0.97	5.83	5.83	3.88			
Vision impaired	0.00	0.97	3.88	7.77	4.85			
Carers/assistants of people with a disability and parents who use a stroller for their child to navigate in the park	0.00	1.94	3.88	13.59	0.00			
Overall	0.97	10.68	33.01	44.66	10.68			

**Figure 1:**
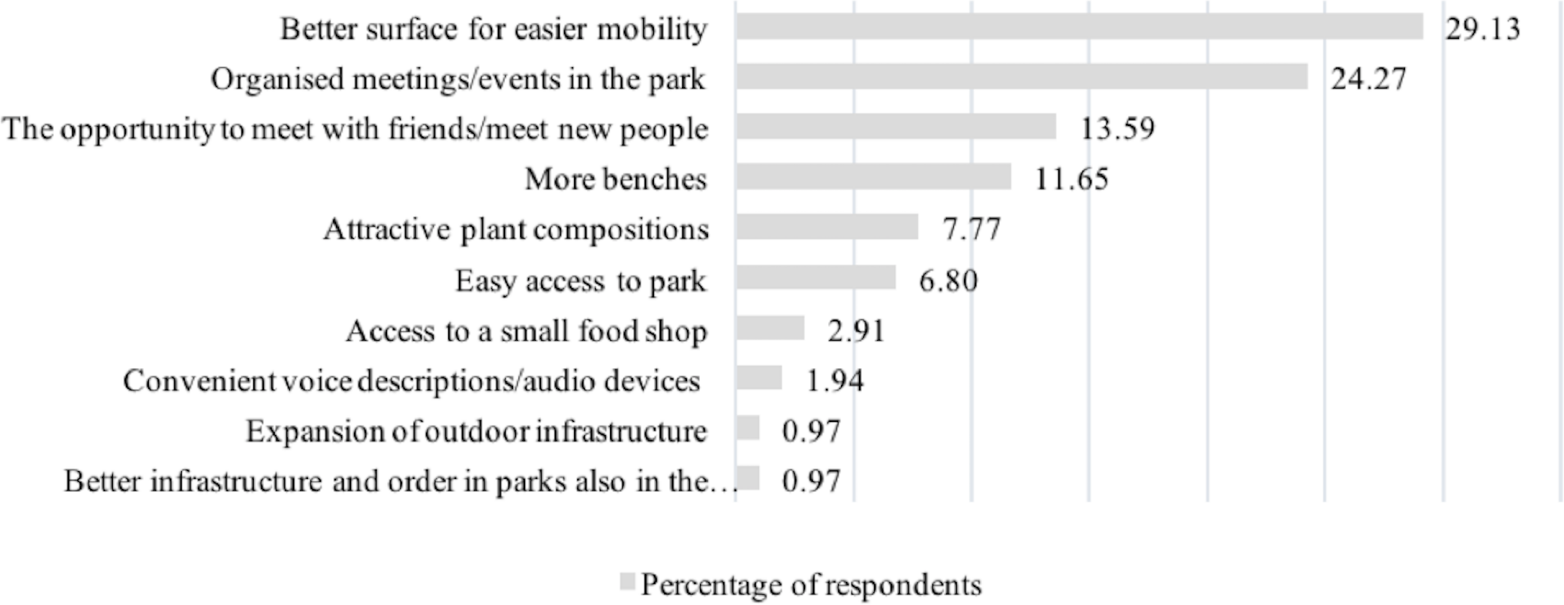
Percentage distribution of answers of all respondents to the question about factors influencing their willingness to stay in the park more often or longer.

When asked what would make them spend more time in the park, the respondents indicated a better surface of footpaths in the first place (29% of all answers). This factor was pointed out by almost 50% of people who use a wheelchair and 29% of blind participants. Twenty-four per cent (24%) of all respondents marked “meetings/events organised in the park” as a factor that impacts park visit frequency. This was the answer of 40% of carers/assistants and parents of children, and 35% of blind contributors. The social aspect was reflected in the answer of 14% of all respondents who indicated the “opportunity of meeting with friends/meeting new people” as a factor influencing their presence in the park. This answer was most often given by vision-impaired respondents. A similar number of all respondents (12%) marked “more benches”. Respectively 8 and 7% of respondents pointed out “attractive plant compositions” and “easy access to the park”. Less than 5% answered the question about the factors influencing their willingness to stay in the park. Three people wrote “access to a small food shop”, and two people added, “need for audio devices to appear in the parks”. Finally, 1% of respondents indicated the “an outdoor gym” and “a better overall infrastructure and order in the park” as factors contributing to their potential presence in the park (Fig. 1).

### Availability and accessibility of parks

The answers to the question about the importance of accessibility of parks showed statistically significant differences among the five groups of respondents. Among people who use a wheelchair, more than 88% considered the accessibility of the park to be “important” or “very important” to them. Only 10% of those surveyed said that it was “rather not important” and “moderately important” to them. For people with a disability, the accessibility to the park was also important. More than 82% indicated that it was “important” or “very important” to them, and only 16% indicated that the availability was “not really important” and “moderately important”. However, the carers/assistants of people with a disability answered differently. More than 62% of the group rated accessibility very high, proving it to be very important to them. Only 10% of the carers/assistants indicated that accessibility of the park was moderately important to them. For 75% of the blind respondents, the availability of parks was important and very important. Almost 43% of the vision impaired group gave the highest ratings to the “very important” option.

When assessing neighbourhood and destination parks in terms of accessibility, the respondents, in general, did not distinguish between these two types. With maximum availability of 515 points, respondents awarded 308 points to destination parks, and 313 points to neighbourhood parks. Among people who use a wheelchair, 20% of the respondents gave 1 to 2 points to destination parks, and 25% rated neighbourhood parks in the same way. The highest ratings: 4 and 5, were awarded to destination parks (more than 50%) and parks with the local designation (more than 47%). In general, the caregiver assessed parks as more accessible than persons from four other groups. The lowest ratings (1 and 2) for destination parks were given by nearly 19% of respondents, while the highest ratings were given to the parks by nearly 52% of them. The vision-impaired participants most often rated both types of parks as moderately accessible, giving them a rating of 3. None of the groups of blind and vision-impaired gave a rating of 5 to any of the parks. The answers given by individual groups of respondents were not statistically significant.

### Barriers and facilities

A statistically significant correlation occurred in the answers to the question “Do you encounter architectural barriers in parks (improper surface. no benches, no slipways, etc.)?”. Among people in wheelchairs, more than 75% said “definitely yes” and “rather yes”, almost 17% said “rather not”, and about 7% expressed no opinion on the issue. Approximately two-thirds of people with a mobility problem (63%) declared that they encountered barriers in the parks; half of them chose “definitely yes”. Every fifth person of the whole group of the respondents said rather not to encountering barriers in parks, and every seventh person had not thought about the issue. Carers/assistants people with a disability and were the only group of respondents in which a majority (60%) marked “difficult to say” and “rather not”, of which every third person declared ”rather not”. Among the blind people, 41% marked “definitely yes”, and “rather yes”. Around 11% stated that they did not encounter architectural barriers in parks (rather not). The most frequently marked answer in the group of vision impaired was “rather yes”. About 17% considered that they did not encounter (rather not) architectural barriers in the parks. Out of the whole research group, almost 37% indicated that they encountered architectural barriers in the park (rather yes), about 30% of the respondents encountered such barriers, and 13% of the respondents were not sure. Nineteen per cent of the respondents marked “rather not”, of which 30% were carers/assistants of people with a disability.

The answers to the next item in the questionnaire made it possible to identify the most burdensome barriers as perceived by the respondents. The results turned out to be statistically significantly depending on the type of disability. The respondents could indicate a maximum of three barriers and add their proposal. The most frequently indicated obstacle was ”inappropriate surface—it was chosen by 50% of the respondents. More than 45% indicated the lack of an appropriate number of benches or seats, and 30% indicated the lack of accessible toilets. Between one fifth and a quarter of all respondents indicated that there was a problem: difficult access to the park, lack of security, lack of program and too high steps.

**Figure 2:**
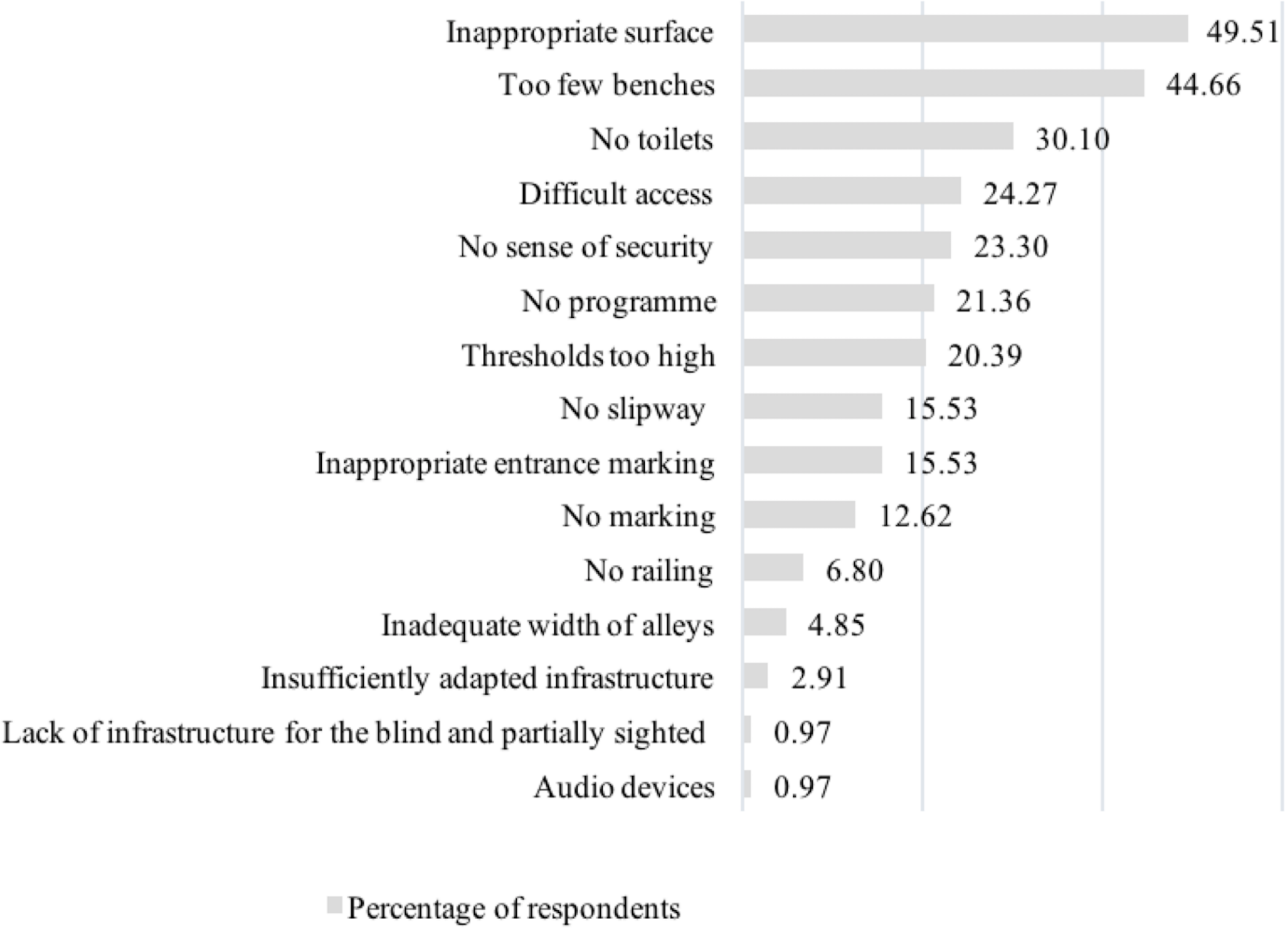
Percentage distribution of answers of all respondents to the question concerning the most burdensome barriers in the parks.

For people in wheelchairs, the improper surface was the nuisance. This answer was given by 69% of the respondents. The next most frequently chosen barrier was “no accessible restrooms for people with a disability” and “no ramps by the stairs or an alternative road adapted for people with reduced mobility”. Thirty-one per cent of the wheelchair users indicated “difficult access to the park (no lifts for underground passages, unmarked pedestrian crossings, etc.)”. “Improper surface” and “too few benches or sits” were identified as the main barriers by even more people with limited mobility than wheelchair users. This was 74 and 68% respectively. To a much lesser extent, they indicated “lack of an adequate park programme” or “difficult access to the park”. Each of these answers appeared in 26% of the group with impeded mobility. Approximately a quarter (26%) of people with limited mobility indicated “lack of an adequate park program” or “reduced access to the park”. Approximately 40% of carers/assistants of people with a disability and indicated “improper surface”, “too few benches or seating places”, “no adequate park program”, and “no sense of security”. The most frequently indicated answers among the blind respondents were: “inappropriate marking of entrances/exits to/from the park” and “no appropriate marking of the park”. They were indicated by 53% of those surveyed. Importantly, none of the other groups marked these answers. Around 20% of the respondents also indicated that they were discouraged by ”improper surface”, “too few benches or seats”, and “difficult access” to the park. One of the respondents added their own answer “lack of audio (voice) information” was an obstacle in parks (Fig. 2).

**Figure 3:**
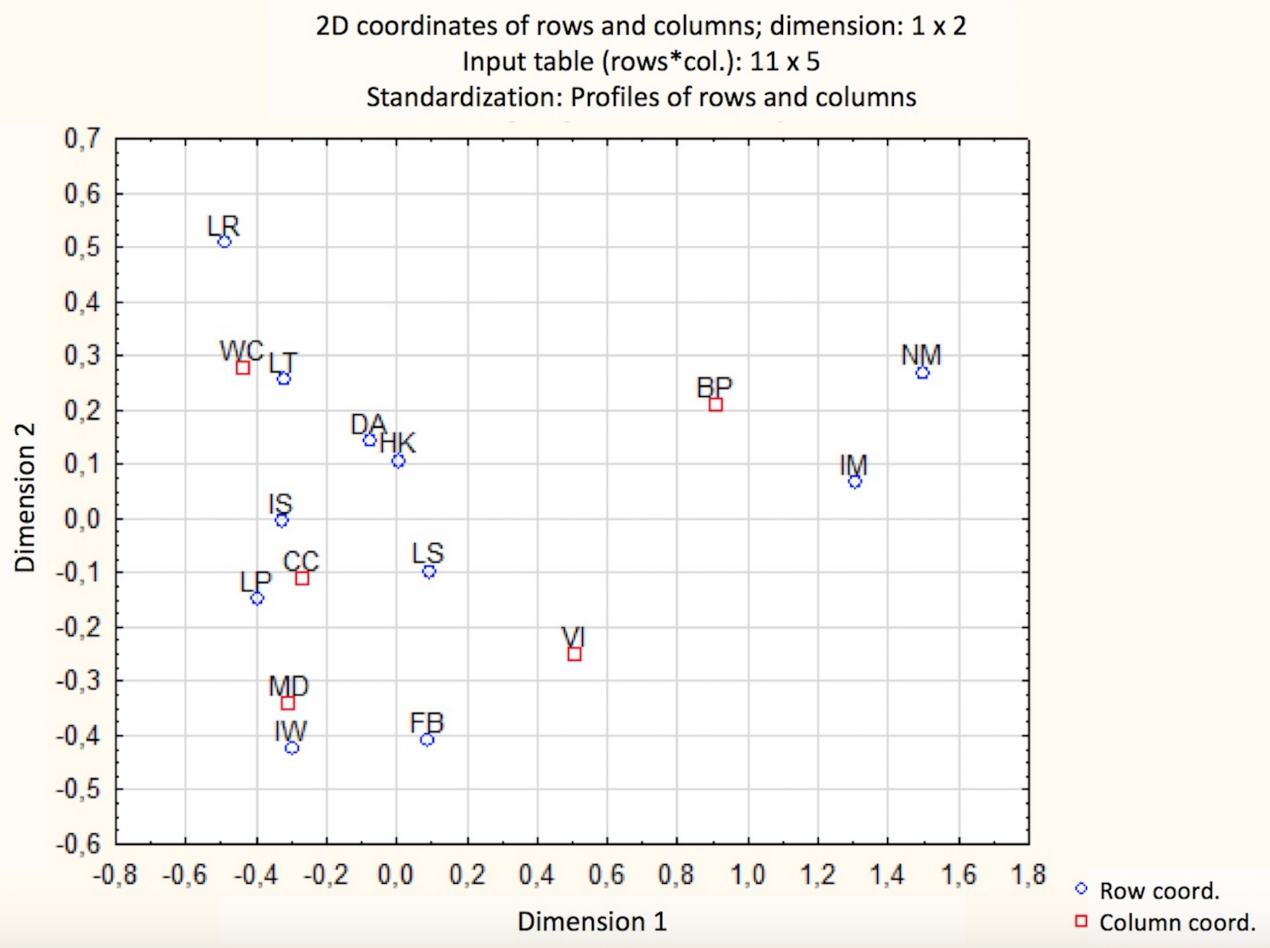
The relationship between the groups of disabilities and the type of barrier (the distance between these elements reflects the strength of the relationship). (LR, lack of ramps; LT, lack of accessible toilets; DA, difficult access to the park; HK, too high thresholds (step slope pads); IS , improper surface; LS, lack of feeling of safety; LP, lack of the park’s programme; IW , improper width of alleys; FB, too few benches; IM, inappropriate park entrance; NM , lack of appropriate park markings; WC, wheelchair users; CC, carers/assistants of people with a disability and parents who use strollers for their children; MD, people with mobility problems; VI , vision impaired; BP, blind).

The analysis of the correspondence (Fig. 3) allowed visualising the relationship presented above. It showed that people with a disability, as well as parents of small children, pointed to the same barriers with a similar frequency. The answers of blind and vision-impaired participants were different. The carers/assistants of people with a disability mainly pointed to lack of the park’s programme (LP) and the improper surface. People who use a wheelchair pointed to the lack of accessible toilets (LT), followed by the lack of ramps (LR), difficult access to the park (DA), too high thresholds (step slope pads) or curbs (Kraw), and improper surface (IS). In the case of the blind and vision-impaired, it is difficult to indicate the most dominant barriers. A slight indication refers to the lack of appropriate park markings (NM), and inappropriate park entrance/exit markings (IM). In the case of the vision impaired respondents, a general lack of feeling of safety (LS), and too few benches (FB) were pointed out as the most considerable difficulties.

### Activities and comfort of movement

**Figure 4:**
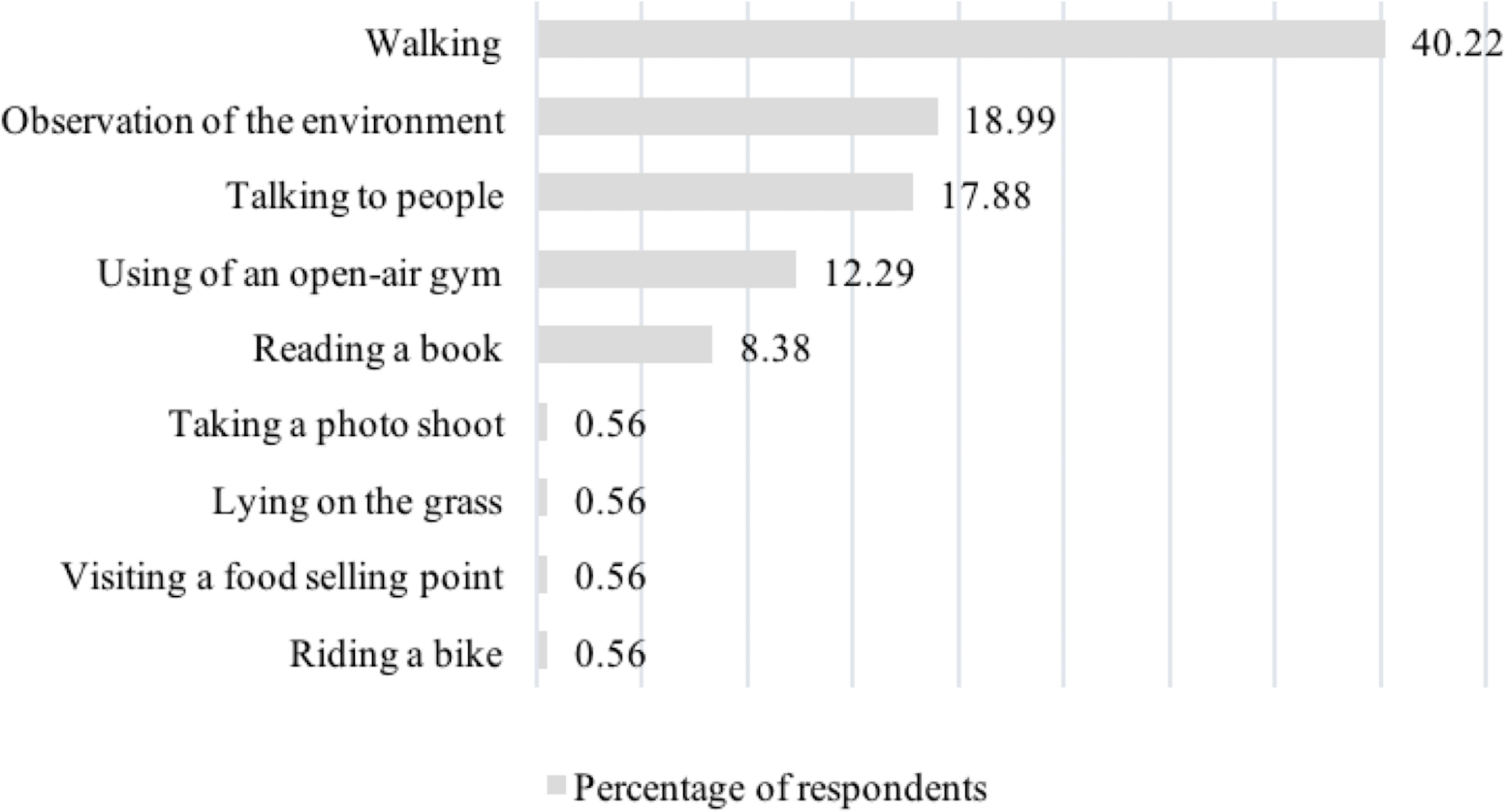
Percentage distribution of answers of all the respondents to the question concerning the most frequently performed activities.

As the most frequent activity, the respondents chose walking (40% of the respondents). Next, they indicated “observing the environment”, and “talking”—these answers were selected by nearly 19 and 18% of respondents. The use of outdoor gyms appeared in 12% of the responses. Several respondents took advantage of the possibility to add their answer, introducing four different activities as the most frequent ones. The most common response among people in a wheelchair was a ride (55%). Only one respondent used the opportunity to add his activity. It was taking photo sessions. People with a disability described their activity mainly as walking. Next, they mentioned observing the surroundings and using the outdoor gym. All the carers/assistants of people with a disability, except one person, indicated that they most often walked in the park, 30% of the answers referred to “having a chat”. One person added a new activity such as “lying on the grass”. Blind and vision-impaired respondents preferred to spend time in the park, walking. In the blind people group, about 30%, indicated using an outdoor gym as the most frequent activity in the park. The vision-impaired group also used “reading a book” and “riding a bike” answers (Fig. 4). The answers given by individual groups of respondents were not statistically significant.

**Figure 5:**
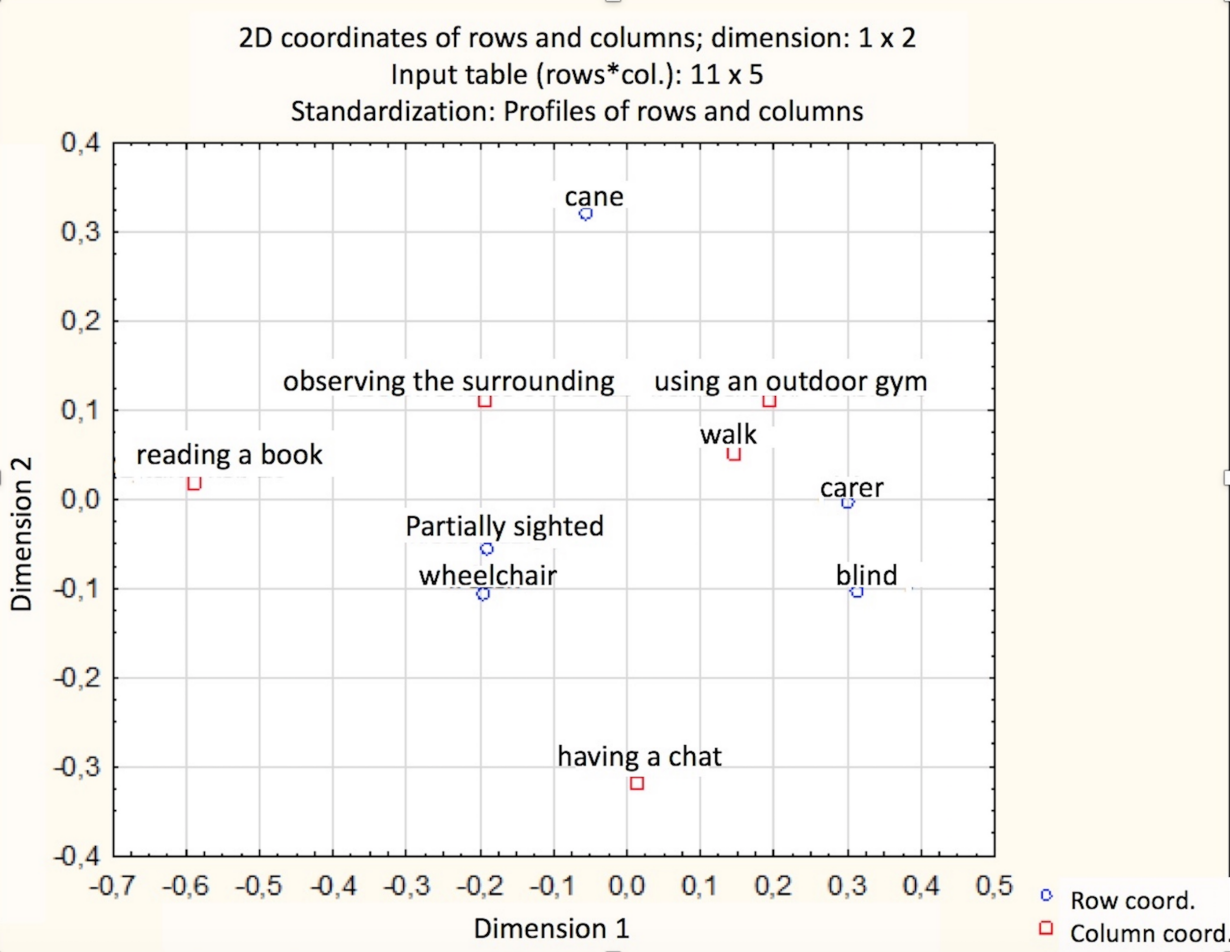
Analysis of the correspondence presenting the relationship between belonging to one of the groups of disabilities and most frequently performed activities (the distance between these elements reflects the strength of the relationship).

The analysis of the correspondence visualised the strength of the relationship between the groups of disabilities and most frequently performed activities. It shows that a similar relationship between activity and disability occurs in the case of vision impaired and people in wheelchairs. A certain similarity can also be observed in the case of blind and carers/assistants of people with a disability and (Fig. 5).

When asked about participation in activities organised in parks, almost 60% of people who use a wheelchair answered negatively. Every third person said, “definitely yes”. A similar number of people with impaired mobility indicated “rather yes” and “rather not”. In turn, 21% answered “definitely yes”, and 16% “definitely not”. Fifty per cent of carers/assistants of people with a disability and declared using additional attractions in the park, with half of them choosing “definitely yes”. Only 10% of respondents from that group declared that it was difficult for them to answer. According to the survey, 47% of blind respondents selected “rather not” when asked about using attractions offered by the park, and about 35% said, “rather yes”. In this group of respondents there was no answer “difficult to say”. 44% Forty-four per cent of vision-impaired said that they use attractions (rather yes) and 33% said rather not. Strong responses prevailed in the overall pool. Only 5% of all the respondents chose the “not sure” option. The answers given by the five groups were not statistically significant (Table 3).

**Table 3:** Differences in participation in the attractions organized in the park (events, meetings, playgrounds for adults, board games, etc.) or usage of the park equipment between separate groups of the respondents.

	**Definitely no**	**Rather not**	**Not sure**	**Rather yes**	**Absolutely yes**	*χ*2	**df**	*p*-value
People who use a wheelchair	4.85	11.65	0.97	1.94	8.74	0.9223767	5	0.9686
People with impaired mobility	2.91	5.83	0.00	5.83	3.88			
Blind	0.97	7.77	0.00	5.83	1.94			
Vision impaired	0.97	5.83	1.94	7.77	0.97			
Carers/assistants of people with a disability and parents who use strollers for their child to navigate in the park	0.97	6.80	1.94	4.85	4.85			
Overall	10.68	37.86	4.85	26.21	20.39			

In response to the question concerning the use of mobile applications facilitating movement in public space, majority of people chose “no, but if I knew about them, I would be happy to use them”. Among people in wheelchairs, 21% declared that even if they knew such applications, they would not benefit from them. In the case of people with reduced mobility, 26% gave the same responses. Among the carers/assistants people with a disability and parents of children, only 15% declared reluctance to use mobile applications that facilitate movement in public space. Every tenth blind person in the survey declared using mobile applications. Among the partially sighted, 22% did not use and did not want to use mobility aids. Furthermore, over 77% of respondents from this group declared willingness to use applications if they are known to them. Among the mobile aids listed were GoogleMaps, JakDojadę. Totupoint, and Seeing Assistant Move. The answers given by particular groups of respondents were not statistically significant.

Finally, the respondents were asked about using assistive technology devices and mobile assistive applications in the parks. The respondents had the opportunity to say what kind of help they used. Among people in a wheelchair, only one person knew such aids and used them. Sixty-five per cent did not know such materials but would be happy to use them. Almost every third person from the group declared unwillingness to use such aids. None of the people with a disability knew the aids, but more than 63% of the group declared their willingness to use the aids if they were available in the parks. Among the caregivers, 45% admitted to knowing and using assistive technology devices and mobile assistive applications, most often mentioning park maps and models. The same percentage of people from the group indicated that if they knew such aids, they would gladly use them. Seventy-six per cent of blind declared their willingness to use the material aids available in the park, and only 18% would not want to use them, even if they were available. One person declared the use of such aids and pointed to touch maps. Among the partially sighted, the majority of people declared their willingness to use assistive technology devices and mobile assistive applications if made available by the park. Every 6th person would not want to use such facilities in the park. Eleven per cent of respondents from the group would not use such an option if the park offered it.

Sixty-four per cent of all the respondents declared willingness to use the assistive technology devices and mobile assistive applications available in the parks if they were available. Twenty-three per cent (the same as in the case of mobile aids) were unwilling and more than 12% already used such aids. The answers to the question about the importance of accessibility of parks showed statistically significant differences among the five groups of respondents.

### Answers vs social characteristics

Responses of people from different groups of disabilities did not differ significantly in terms of age, gender, and education.

## Discussion

The generalizability of the results is limited by a relatively small number of survey participants, considering all other city residents who may encounter mobility problems due to their own motor or sensory disability. The methodological choices were constrained by no sampling list. No random sampling was possible and thus it was difficult to select a proportional sample. However, the so-called good mix of representatives of different population groups was applied that allowed covering a wide spectrum of mobility difficulties in this study.

It is beyond the scope of this study to set the general rules for universal urban park design. However, the data obtained allow proposing recommendations and guidelines for new builds and large-scale parks. *A posteriori* studies among people with mobility difficulty are still rare. The guidelines resulting from ergonomics that decides pathway dimensions based on wheelchair parameters are widely known. However, the user experience assessment of the park quality by people with mobility difficulty has brought a lot of new information. An important obstacle, indicated by the largest number of respondents, is the inappropriately selected material for the pathways. Uneven sand or gravel paths were indicated as one of the main inconveniences. Accessible communication (touch maps, etc.) and physical markers and high contrast borders are required for some of the cohorts in the study. These elements are not included in current standards for park design. Study results show that, in most cases, outdoor furniture or information systems located in Warsaw city parks do not meet the necessary conditions for people with mobility difficulty. Moreover, assessed responses indicate that the infrastructure often fails to meet the legal requirements imposed by the Construction Law Act and the relevant Regulation of the Minister of Infrastructure.

The results of the study revealed some differences in the perception of the accessibility, availability and use of parks among people from the five different groups with limited mobility. Urban parks are generally positively valued by the respondents. The majority of them revealed that they liked to spend time in a park, with almost no negative answers at all. Apart from vision impaired, in all groups, the dominant answer was “definitely yes”. The answer to the second question was also some kind of confirmation of the answer to the first question. The respondents presented themselves as people who were frequent park users (despite obvious difficulties related to movement). What is particularly interesting, almost one-fifth of the respondents revealed that they were in the parks daily, and almost one third were in the parks several times a week. This result proves that benefits of nature (also shown in other studies (Corazon et al., 2019; Suchocka, Jankowski & Błaszczyk, 2019)), and their need to be surrounded by the natural environment (Wojnowska-Heciak, 2019; Wojnowska-Heciak et al., 2020). This trend can also be seen among the residents of other countries; in Denmark, 43 per cent of the respondents reported visiting green spaces every day and 91 per cent at least once a week (Schipperijn et al., 2010). Our study did not involve people from the able-bodied population (as the comparison of these two groups was not the subject of the study, it was the differences within people with a disability and impeded mobility), so it is impossible to refer to the results of the studies by Stigsdotter Stigsdotter, Corazon & Ekholm (2017) and Corazon Corazon et al. (2019), which showed that individuals with mobility disabilities visited green spaces less frequently than people without them.

More than half of the respondents revealed that they usually spent about an hour in the park, and almost one third about 2–3 h. Time spent in a park is undoubtedly related to the distance to it, which to some extent found its reflection in the answers to the preferred type of parks. In the case of this question, significant statistical differences were revealed, with one-third of the responses from all groups indicating spending time only in parks of local significance—neighbourhood parks, and almost 45% more often in parks of this type. People who use a wheelchair constituted the largest group out of the five examined both in terms of the choice of destination park and the length of time spent in the park—visiting a destination park may be a goal in itself, which is why it involves an extended stay.

Preference for neighbourhood parks may indicate that the key issue for the respondents is the distance (proximity), followed by time availability. These factors were indicated as leading to increased park use in the research discussed in the introduction section (Bedimo-Rung, Mowen & Cohen, 2005; Byrne & Wolch, 2009; Giles-Corti et al., 2005; Reyes, Páez & Morency, 2014; Liang, Chen & Zhang, 2017; Wüstemann, Kalisch & Kolbe, 2017; Feng et al., 2019). Additionally, it should be underlined, that in many cases, the increase in distance entails an increase in barriers (mainly physical, but also psychological) to be overcome. Therefore, neighbourhood parks seem to be particularly important to people with a disability.

When assessing the accessibility of neighbourhood and destination parks, the respondents did not generally differentiate between the two types. Just over half awarded the highest ranks (4 and 5) to destination parks, and slightly fewer gave the same ranks to the neighbourhood parks. About 22% of respondents defined neighbourhood parks as inaccessible (ranks 1 and 2), with 18% of respondents saying the same with respect of destination parks. Although the results of the study did not show statistically significant differences between the groups, it is worth noting that the blind and vision-impaired participants did not give the highest ratings at all. This is probably due to higher requirements as for the park facilities (e.g., touch maps, audio devices). By showing a preference for visiting neighbourhood parks, respondents declare that it is park proximity that plays a key role in determining the decision of park use. In this context, the distribution of parks within a city seems particularly important. The issue, strongly tied with environmental justice, was addressed in the study analysing access to a Guo et al. (2019) park for the elderly (and thus posing problems with mobility).

The respondents indicated clear factors that could contribute to more frequent park use. Interestingly, the two main ones were completely different in character. The first one (29%) was a physical factor: a better surface to facilitate movement. As the second one (24%), the social factor was pointed out: meetings/events organised in parks. The third reason for more frequent visits to a park (13% of responses) was the opportunity to meet people. Distribution of the answers indicates that the issues of physical accessibility are of the same importance as socio-psychological ones. As indicated by numerous studies, people with limited mobility are exposed to social exclusion, which, we assume, is reflected in the results of this survey. According to data obtained independently by different researchers, partially sighted persons may be the group particularly seeking for social relations (Vuletić, Šarlija & Benjak, 2016; Kasiram & Subrayen, 2013).

In the group of questions concerning the perceived physical barriers, the answers of the respondents were presented with statistical differences. Around 67% of the respondents gave affirmative answers (rather yes and definitely yes), however, among the “definitely yes” answers only a small percentage belonged to carers/assistants od people with a disability and parents of children. The groups of people who answered positively in the largest proportion (definitely yes) were the blind (41%) and those in a wheelchair (45%). At the same time, these two groups had only a few per cent share in the “not sure” answer. Interestingly, none of the respondents gave a “definitely not” answer. Differences in the answers show that the perception of barriers by carers/assistants is different from the perception of people with a disability. To some extent, and under certain circumstances, they experience the necessity to overcome them. The results of the survey are consistent with those of other researchers, including Penchansky & Thomas (1981), who evaluated 21 public parks (neighbourhood and destination ones) in metropolitan cities of New Zealand. None of them met the criteria of accessibility and usability for persons with a disability.

By listing the most significant barriers to mobility, the respondents also showed statistically significant differences in their answers, which is obviously related to the type of disability and thus - barriers to overcome. The majority of respondents considered inadequate surface (50%) to be the main barrier, followed by too few benches and no toilet. The unsuitable surface was also mentioned as one of the main problems in Perry et al. (2018a). It is worth mentioning, however, that in the responses of blind and vision-impaired people, the dominant barrier was not indicated. It seems that in adapting parks to the needs of people with a disability, the needs of persons with vision impairment are often left unnoticed.

The activities indicated by the respondents can be considered “typical”. The first place was given to walking, followed by “observing the environment”, and “talking. The differences between the groups and the answers of the respondents were not taken over. It should be noted, however, that the main activities performed in the park are both physical (and therefore indicate the association between parks and physical activity (Schipperijn et al., 2017), and social. Such a distribution of the answers suggests, again, that parks do constitute an important place of building and maintaining social relations for persons with limited mobility. However, when answering the question about participation in events organised in the park (or use of its attractions), almost half of the respondents admitted that they did not (definitely not or rather not) take part in them (with a smaller number of people declaring that they did). Since the aim of the study was not to explain the mechanisms but to assess the existing condition, and the respondents were not asked about the reasons for participation or lack of participation, it can only be assumed that this is related to both personal factors (such as the preference of crowd avoidance) or enticed physical barriers.

Most of the respondents revealed that they did not use mobility applications that would make it easier for them to get around in parks but would be willing to use them. Similar results were obtained from responses about using assistive technology devices and mobile assistive applications in the parks. The results of the study indicate that the use of tools supporting comfort during a park visit is unlikely to be associated with seeing oneself as “different, unsuitable”. It is thus difficult to see an analogy with the results of the Seeland & Nicolè study (2006), which showed that adapting park space to the needs of people with a disability is associated with the issue of feeling stigmatised. Information and Communication Technologies (ICT) seem to be a tool that could be used to enhance park users with different needs, e.g., in promoting active recreation or even involve them in urban games (an idea of Playable City) (Kimic, Maksymiuk & Suchocka, 2019; Suchocka, Kimic & Widaj, 2019) and may extend an offer for users looking for events in the park or social contacts. That approach could help them to shorten the social distance, decrease the feeling to be stigmatised or excluded and bring more relaxation.

## Conclusions

In conclusion, people representing five different groups of people with limited mobility in most part present a similar perception of park accessibility. The differences concern preferences for local and destination parks, as well as encountered architectural barriers and the use of assistive technology devices and mobile assistive applications. The answers of the respondents revealed that the accessibility of parks is important for them. They also presented themselves as regular visitors to parks, especially local parks. Therefore, all activities related to urban planning must enable equal access to parks, including local ones. This will allow not only for the rehabilitation of people with a disability but also for their greater social inclusion of other groups facing mobility difficulties.

Further research on the park use should cover identification of mechanisms that determine the functioning of people with limited mobility in urban space, particularly in parks. Solutions such as ITC could be used for users’ needs recognition and park attractiveness improvement. It also seems important that all activities related to the (re)construction of parks should be carried out with the participation of people from different groups of mobility difficulties as their needs and limitations are different and sometimes may simply not be recognised by body-able designers.

##  Supplemental Information

10.7717/peerj.10570/supp-1 Supplemental Information 1 Raw data.

10.7717/peerj.10570/supp-2 Supplemental Information 2 Survey in English.

10.7717/peerj.10570/supp-3 Supplemental Information 3 Survey in Polish.

## Additional Information and Declarations

### Data Availability

The following information was supplied regarding data availability: Raw data and the survey are available in the Supplementary Files.
