# Supplementary material for: Quality of urban parks in the perception of city residents with mobility difficulties
Source: PeerJ. 2020 Dec 18;8:e10570. doi: 10.7717/peerj.10570 (PMC7751420; doi:10.7717/peerj.10570)
Supplement: Supplemental Information 2 [file peerj-08-10570-s002.docx]

**Survey questionnaire**

The survey concerns the accessibility of green areas for people with reduced mobility and is addressed to these people. The questionnaire consists of 17 questions and takes no more than 5 minutes to complete.

In the first part of the questionnaire there are mostly single-choice questions. Question nr 10 allows for a maximum of 3 answers. There are also questions where the answer should be assessed on a scale of 1-5. The second part of the questionnaire serves to determine the respondent's profile. If the questionnaire is filled in by the Carer of a disabled person, please enter the data of the ward.

Part one

1. Do you like spending your free time in the park?

- Absolutely yes
- Rather yes
- Not sure
- Rather no
- Definitey no

2. How often do you visit the park?

- Everyday
- Several times a week
- Several times a month
- Several to over a dozen time a year
- Never

3. How much time do you usually spend in the park?

- Up to 30 minutes
- Around 1 hour
- Around 2 hours
- More than 3 hours

4. What do you usually do in the park?

- Walking
- Talking to people
- Observation of the environment
- Reading a book
- Using of an open-air gym
- Other………………………………………………………………………………………

5. What would make you spend more time in the park?

- More benches
- Organized meetings/events in the park
- The opportunity to meet with friends/new people
- Better surface for easier mobility
- Easy access to park
- Attractive plant compositions
- Other………………………………………………………………………………………

6. Do you prefer spending your free time in destination parks (e.g. Royal Łazienki Park, Wilanów Palace and Park ) or in neighbourhood ones (e.g. parks close to home, pocket parks). ?

- I only spend time in destination parks
- I spend time in both types of parks, but more often of in destination ones
- I only spend time in neighbourhood parks
- I spend time in both types of parks, but more often in neighbourhood ones

I spend as much time in both types of parks

7. How important is the park availability to you? Mark your answer on a scale of 1-5, 1 – not important, 5 – very important.

1 2 3 4 5

not important □ □ □ □ □ very important

8. How do you perceive the availability of the two types parks (1 - inaccessible, 5 completely accessible).

1 2 3 4 5

Destination parks □ □ □ □ □

Neighbourhood parks □ □ □ □ □

9. Do you encounter architectural barriers in parks (improper surface, no benches, no slipways, etc.)?

- Absolutely yes
- Rather yes
- Not sure
- Rather no
- Definitey no

10. What kind of barriers are the most inconvenient for you? Please choose up to 3 answers.

- Inappropriate surface
- Inappropriate entrance marking
- Too few benches
- No markings
- No programme
- No sense of security
- Difficult access to the park
- No tolilets
- No slipway
- No railing
- Thresholds too high
- Inadequate width of alleys
- Other………………………………………………………………………………………

11. Do you take part in the attractions organized in the park (events, meetings, playgrounds for adults, board games, etc.) or usage of the park equipment between separate groups of the respondents.

- Absolutely yes
- Rather yes
- Not sure
- Rather no
- Definitey no

12. Do you use mobile applications to facilitate movement in public spaces?

- No, and I would not use them even if they were accessible
- No, but if I knew about them, I would be happy to use them
- Yes, I do (please write the name of them)

………………………………………………………………………………………

13. Do you use the assistive technology devices and mobile assistive applications available in the parks (maps, mock-ups, navigators, etc.)?

- No, and I would not use them even if they were accessible
- No, but if I knew about them, I would be happy to use them
- Yes, I do (please write the name of them)

…………………………………………………………………………………………

Part two

1. Gender

- Woman
- Man
- I do not want to reveal it

2. How old are you?

- 18 -29 years old
- 30- 39 years old
- 40 - 49 years old
- 50 – 59 years old
- <60 years old

3. Education

- Basic
- Vocational
- High school
- Higher
- Student

4. What makes it difficult for you to move?

- I am a wheelchair user
- I have mobility problems, but I do not use a wheelchait
- I am blind
- I am partially sighted
- I am a carer of a disabled person/I am a parent of a young child

Thank you very much for completing the questionnaire and participating in the survey!
