# Supplementary material for: Quality of urban parks in the perception of city residents with mobility difficulties
Source: PeerJ. 2020 Dec 18;8:e10570. doi: 10.7717/peerj.10570 (PMC7751420; doi:10.7717/peerj.10570)
Supplement: Supplemental Information 3 [file peerj-08-10570-s003.docx]

Kwestionariusz

Badanie dotyczy dostępności terenów zielonych dla osób o ograniczonej sprawności ruchowej i jest skierowane do tych osób. Kwestionariusz składa się z 17 pytań, a jego wypełnienie nie zajmuje więcej niż 5 minut. W pierwszej części kwestionariusza znajdują się głównie pytania jednokrotnego wyboru. Pytanie nr 10 dopuszcza maksymalnie 3 odpowiedzi. Są też pytania, na które odpowiedź należy oceniać w skali 1-5. Druga część kwestionariusza służy do określenia profilu respondenta. Jeżeli kwestionariusz wypełnia Opiekun osoby niepełnosprawnej prosimy o podanie danych podopiecznego.

Część pierwsza

1. Czy lubisz spędzać wolny czas w parku?

□ Absolutnie tak

□ Raczej tak

□ Nie jestem pewien

□ Raczej nie

□ Zdecydowanie nie

2. Jak często odwiedzasz park?

□ Codziennie

□ Kilka razy w tygodniu

□ Kilka razy w miesiącu

□ Kilka do kilkunastu razy w roku

□ Nigdy

3. Ile czasu zazwyczaj spędzasz w parku?

□ Do 30 minut

□ Około 1 godziny

□ Około 2 godzin

□ Ponad 3 godziny

4. Co zwykle robisz w parku?

□ Chodzenie

□ Rozmowa z ludźmi

□ Obserwacja środowiska

□ Czytanie książki

□ Korzystanie z siłowni na świeżym powietrzu

□ Inne ………………………………………………………………………………………

5. Co sprawiłoby, że spędziłbyś więcej czasu w parku?

□ Więcej ławek

□ Organizowane spotkania / imprezy w parku

□ Możliwość spotkania się z przyjaciółmi / nowymi ludźmi

□ Lepsza powierzchnia dla łatwiejszej mobilności

□ Łatwy dostęp do parku

□ Atrakcyjne kompozycje roślinne

□ Inne ………………………………………………………………………………………

6. Wolisz spędzać wolny czas w parkach docelowych (np. Łazienki Królewskie, Pałac i Park w Wilanowie) czy też w sąsiedztwie (np. Parki blisko domu, kieszonkowe).?

□ Przebywam tylko w parkach docelowych

□ Spędzam czas w obu typach parków, ale częściej w docelowych

□ Przebywam tylko w okolicznych parkach

□ Przebywam w obu typach parków, ale częściej w okolicznych

Spędzam tyle samo czasu w obu typach parków

7. Jak ważna jest dla Ciebie dostępność parku? Zaznacz swoją odpowiedź w skali 1-5, 1 - nieważne, 5 - bardzo ważne.

1 2 3 4 5

nieważne □ □ □ □ □ bardzo ważne

8. Jak oceniasz dostępność dwóch typów parków (1 - niedostępne, 5 całkowicie dostępne).

1 2 3 4 5

Parki docelowe □ □ □ □ □

Okoliczne parki □ □ □ □ □

9. Czy napotykasz bariery architektoniczne w parkach (niewłaściwa nawierzchnia, brak ławek, pochylni itp.)?

□ Absolutnie tak

□ Raczej tak

□ Nie jestem pewien

□ Raczej nie

□ Zdecydowanie nie

10. Jakie bariery są dla Ciebie najbardziej uciążliwe? Wybierz maksymalnie 3 odpowiedzi.

□ Niewłaściwa powierzchnia

□ Niewłaściwe oznakowanie wejścia

□ Za mało ławek

□ Brak oznaczeń

□ Brak programu

□ Brak poczucia bezpieczeństwa

□ Utrudniony dostęp do parku

□ Brak toalet

□ Brak pochylni

□ Brak poręczy

□ Progi zbyt wysokie

□ Niewystarczająca szerokość alejek

□ Inne ………………………………………………………………………………………

1. Czy bierzesz udział w atrakcjach organizowanych na terenie parku (imprezy, spotkania, place zabaw dla dorosłych, gry planszowe itp.) Lub korzystasz z wyposażenia parku pomiędzy poszczególnymi grupami respondentów.

□ Absolutnie tak

□ Raczej tak

□ Nie jestem pewien

□ Raczej nie

□ Zdecydowanie nie

12. Czy korzystasz z aplikacji mobilnych, aby ułatwić poruszanie się w przestrzeni publicznej?

□ Nie, i nie użyłbym ich, nawet gdyby były dostępne

□ Nie, ale gdybym wiedział o nich, chętnie z nich skorzystałbym

□ Tak, tak (proszę podać ich imiona)

……………………………………………………………………………………………

13. Czy korzystasz z urządzeń technologii wspomagających i mobilnych aplikacji wspomagających dostępnych w parkach (mapy, makiety, nawigatory itp.)?

□ Nie, i nie użyłbym ich, nawet gdyby były dostępne

□ Nie, ale gdybym wiedział o nich, chętnie z nich skorzystałbym

□ Tak, tak (proszę podać ich imiona)

…………………………………………………………………………………………

Część druga

1. Płeć

□ Kobieta

□ Man

□ Nie chcę tego ujawniać

2. Ile masz lat?

□ 18 - 29 lat

□ 30-39 lat

□ 40 - 49 lat

□ 50 - 59 lat

□ <60 lat

3. Edukacja

□ Podstawowe

□ Zawodowe

□ Szkoła średnia

□ Wyżej

□ Uczeń

4. Co utrudnia Ci poruszanie się?

□ Jestem na wózku inwalidzkim

□ Mam problemy z poruszaniem się, ale nie używam wózka inwalidzkiego

□ Jestem ślepy

□ Jestem niedowidzący

□ Jestem opiekunem osoby niepełnosprawnej / jestem rodzicem małego dziecka

Bardzo dziękujemy za udział i wypełnienie ankiety!
